# Microbial Contamination in Urban Marine Sediments: Source Identification Using Microbial Community Analysis and Fecal Indicator Bacteria

**DOI:** 10.3390/microorganisms13050983

**Published:** 2025-04-25

**Authors:** Ellinor M. Frank, Carolina Suarez, Isabel K. Erb, Therese Jephson, Elisabet Lindberg, Catherine J. Paul

**Affiliations:** 1 Water Resources Engineering, Department of Building and Environmental Technology, Lund University, P.O. Box 118, SE-221 00 Lund, Sweden; 2 Sweden Water Research, Fabriksgatan 2B, SE-222 35 Lund, Sweden; 3 Applied Microbiology, Department of Chemistry, Lund University, P.O. Box 124, SE-221 00 Lund, Sweden; 4 City of Helsingborg, Department of City Planning, Järnvägsgatan 22, SE-252 25 Helsingborg, Sweden

**Keywords:** *Escherichia coli*, sediment microbial community, source tracking, 16S rRNA gene, anthropogenic influences

## Abstract

We investigated the presence of the fecal indicator bacteria *Escherichia coli*, and other taxa associated with sewage communities in coastal sediments, near beaches with reported poor bathing water quality, focusing on the influence of effluent from a local wastewater treatment plant (WWTP) and combined sewer overflows (CSO). Using a three-year dataset, we found that treated wastewater effluent is a significant source of sewage-associated taxa and viable *E. coli* in the sediments and that no seasonal differences were observed between spring and summer samples. CSO events have a local and temporary effect on the microbial community of sediments, distinct from that of treated wastewater effluent. Sediments affected by CSO had higher abundances of families Lachnospiraceae, Ruminococcaceae, and Bacteroidaceae. Sewage releases may also impact the natural community of the sediments, as higher abundances of marine sulfur-cycling bacteria were noticed in locations where sewage taxa were also abundant. Microbial contamination at locations distant from known CSO and treatment plant outlets suggests additional sources, such as stormwater. This study highlights that while coastal sediments can be a reservoir of *E. coli* and contain sewage-associated taxa, their distribution and potential origins are complex and are likely not linked to a single source.

## 1. Introduction

Deterioration of coastal water quality is a growing concern in many urbanized areas [1,2,3]. Industrial discharges, wastewater treatment plant (WWTP) effluents, untreated stormwater, and runoff can contribute to the spread of substances and microorganisms that negatively affect the aquatic environment [4]. Alongside an impact on marine life [5], water quality deviations can result in public health hazards associated with poor bathing water quality [6,7], such as gastrointestinal illness, ear ailments, and urinary tract infections [6]. To reduce risks to the public from contact with recreational waters, it is essential to monitor microbial water quality, which currently relies largely on the use of the fecal indicator bacteria (FIB) *Escherichia coli* and *Enterococcus* spp.

Viable *E. coli* and other pathogenic microorganisms such as *Campylobacter* spp., *Salmonella* spp., *Vibrio* spp., *Cryptosporidium* spp., *Giardia* spp., *Shigella* spp., *Enterococcus* spp. are found in coastal sediments [8,9,10,11,12,13]. Sediments are generally seen as a favorable environment for FIB, as their abundance is often higher there than the overlaying water [11,14]. The formation of biofilm on the surface of the sediment [15] and nutrient availability [16] increase bacterial survival and could explain their high abundance. With resuspension from sediments, FIB and other pathogenic bacteria might enter the water column and eventually reach recreational beaches, directly posing a risk to human health [17].

Understanding FIB contamination in water thus requires analysis of the sediments as a potential reservoir [9,13,18] and determination of their potential sources. The anthropogenic impact (such as from wastewater) on the microbial community in sediments has been shown using both 16S rRNA [9,19,20] and qPCR methodology [21,22]. In 2024, Frank and colleagues also proposed the use of a curated taxa collection for application in source tracking, together with 16S rRNA sequencing [9]. These methods can be used for source tracking, where analysis and cross-comparison of the taxonomic groups associated with known emission sources and those present in the assessed media facilitate determining the source influence, e.g., WWTP effluent, stormwater [23], untreated sewage during combined sewer overflow (CSO) [24], agricultural runoff [25], and non-point sources such as wild birds and domestic animal feces [26]. For CSOs, their impact on microbial water quality has been explored [27,28], but less is known about their effect on the sediment microbial community, particularly for coastal marine sediments.

In previous studies of the same area, sewage-associated bacterial taxa abundance was found to increase with higher *E. coli* concentration, and the sewage taxa abundance decreased with a longer distance from the city’s treated sewage effluent point [9]. Large genotypic diversity in the strains of viable *E. coli* and differences in their potential virulence were also observed [29]. However, it remains unclear whether all viable *E. coli* in the analyzed sediments originated from the treated wastewater effluent. As both previous studies were limited to a single sampling occasion, investigation and understanding of possible temporal or seasonal effects in the microbial communities of the sediments and the quantified viable *E. coli* were not possible.

In this study, microbial communities of coastal sediments at Helsingborg (Sweden) were investigated over a period of three years using quantification of viable *E. coli* and V3–V4 region of 16S rRNA gene sequencing. Our goal was to examine shifts in the microbial community and *E. coli* concentrations and how those could be linked to environmental parameters, as well as assessing the potential impact of microbial contamination by treated and untreated wastewater on these sediments. In particular, we were interested in determining if sources of contamination other than the treated effluent were impacting this area, such as CSOs and stormwater. This was carried out by (1) sampling during a three-year period to allow studying potential dynamics and identify possible intermittent sources of contamination, (2) expansion of the taxonomic groups in the source tracking library previously used [9], aiming to differentiate treated and untreated releases of sewage, and (3) microbial core community analysis, to identify taxa that are unique to one or more samples and which may better reflect the impact of contamination from point sources.

## 2. Material and Methods

### 2.1. Study Site and Sampling

Sampling locations were planned at varying distances to the WWTP outlet, CSO outlet, and stormwater outlet along a four-kilometer coastline of the city of Helsingborg, Sweden. The treatment steps performed in this WWTP are roughing filtration, mechanical purification in parallel pre-sedimentation basins, biological phosphorus and nitrogen purification in activated sludge, and final sedimentation with subsequent sand filtration [30].

In the study site, a halocline is present, and the salinity gradient may vary from 10 to 30 PSU, sometimes in less than two meters of depth. The vertical position of the halocline usually occurs around 10–15 m depth. During the summer seasons, the inorganic nitrogen concentrations are less than 0.5 µmol/L [31].

Marine sediment samples were collected on six occasions: 19 March 2019, 13 August 2019, 17 March 2020, 25 September 2020, 22 March 2021, and 24 August 2021. Sediment samples were retrieved using a core sampler (i.d. 15 cm); the top first cm was collected into a 50 mL sterile polypropylene tube and kept on ice during transit. Sample names are identical to the format used in Frank et al., 2024 [9], indicating the cardinal direction and distance to the wastewater outlet (WWO). The last letter in the sample name denotes when sampling was undertaken, where A are samples from 19 March 2019 and F samples from 24 August 2021.

The sediment type of the area varies with distance; sediments located less than 1217 m from the wastewater outlet (WWO) are very fine-grained, and the sediments between 1217–1240 m north of WWO are fine-grain sand, which is due to an artificial sand beach located straight east of these samples, and the sediments >1917 m north of WWO have larger grains and are heavily mixed with crushed blue mussel shells. Sampling further north (>3340 m) was not possible due to the ocean floor containing large rocks. Sampling further west into the Öresund was not possible due to the border with Denmark. Sampling between 372 and 1217 m north of WWO was not possible because of the passage of ferry lines.

### 2.2. Sample Preparations and Viable E. coli Quantification

Prior to analysis, sediments were homogenized in a petri dish, and 10 mL was transferred into a new 50 mL sterile polypropylene tube (for Colilert18 analysis (IDEXX, Westbrook, ME, USA)). A replicate sample of 6 mL was transferred into cryotubes and stored at −80 °C freezer prior to further processing for DNA sequencing. In the tube with 10 mL of the material, Milli-Q water (MilliporeSigma, Burlington, MA, USA) was filled to a volume of 30 mL. For samples collected on 25 September 2020, 22 March 2021, and 24 August 2021, duplicate subsamples were prepared for Colilert18 analysis.

Sample tubes were then rocked for 18 h at 5–6 °C and then left to settle for one hour. 10 mL of the liquid was decanted. This liquid, containing the resuspended bacteria from the sediments, will provide the most probable number (MPN) per volume unit in the Colilert18 analysis. This is appropriate to use in this study due to the varying moisture content and density of the sediment samples (rather than using per weight unit). In the 19 March 2019 samples, the collected liquid was transported to the VASYD laboratory (Malmö, Sweden) for Colilert18 analysis (see Frank et al., 2024 [9]). In the 13 August 2019 and 17 March 2020 samples, the liquid was diluted as a two-dilution series, yielding a result in the unit of MPN/10 mL sediments). In the 25 September 2020, 22 March 2021, and 24 August 2021, the two duplicates were added together, then diluted as a two-dilution series, yielding a result in the unit of MPN/20 mL sediments). For comparability and unit consistency, the results corresponding to the last three samplings were adjusted to MPN/10 mL sediments. The Colilert18 analysis was performed following the manufacturer’s standard protocol (IDEXX, ME, USA). The Colilert18 method quantifies *E. coli* that are viable (alive and culturable). Descriptions of the samples, including GPS coordinates, distance to WWO, depth the sediments were collected, *E. coli* concentration, season, and year can be found in Table S1. The results from the Colilert18 analysis in Table S1, as well as in the main text, are written in the unit MPN/100 mL, which is the mean of the resulting undiluted concentrations from the dilution series (containing sediments + sterile water). This unit is the same as if the concentration had been written as MPN/10 mL sediments.

### 2.3. DNA Extraction and 16S rRNA Sequencing

In total, sixty-one individual sediment samples were used for DNA extraction. The sediments were retrieved from cryotubes stored in the freezer and thawed prior to analysis. In three of these, DNA was extracted twice, thus yielding three technical replicates (samples E227_D_2, S1_D_2, and W187_D_2). Two negative extraction control samples were generated by using two DNA extraction kits without any addition of sediments. DNA extraction was performed using the FastDNA Spin Kit for Soil (lot no. 136008; MP Biomedicals, Santa Ana, CA, USA). The manufacturer protocol was followed, using the following modifications: centrifuging at 14,000× *g* was extended to 15 min, 700 µL (instead of 600 µL) of the binding matrix–supernatant mixture was transferred to the SPIN filter, and the binding matrix was always resuspended in 100 µL DES, and always followed by 5 min of incubation at 55 °C.

Isolated DNA was sent to a commercial laboratory (Novogene, Cambridge, UK) for DNA sequencing of the V3-V4 region of the 16S rRNA gene with Illumina NovaSeq PE250 (2 × 250 bp read setup) (Illumina, San Diego, CA, USA), primer pair 341F/806R [32,33], and a sequencing depth of 100 kb raw reads. Initial quality filtering and trimming (removed primer and barcode) was performed by Novogene.

### 2.4. ASV Analysis

Sequencing data were generated in FASTQ format, which stores DNA sequences and their corresponding quality scores [34]. FASTQ files were processed using the programming language R (version 4.4.1) [35] in RStudio (version 2023.09.1.494) environment [36]. The FASTQ files were imported into the RStudio environment, where denoising and sample inference were carried out using the DADA2 (package version 1.30.0) [37]. Learning of error rates was undertaken on the first 10^7^ base pairs (bp) for forward and reverse reads separately, and amplicon sequence variants (ASV) were generated with the pseudo-pooling mode. ASVs shorter than 350 bp and longer than 500 bp were discarded (in accordance with Rausch et al., 2019 [38]) using the janitor package (version 2.2.0) [39]. Chimeric sequences were removed using the removeBimeraDenovo-function using argument method = “consensus”. A DADA2 formatted GTDB-database down to genus level was used for taxonomy assignment (GTDB_bac120_arc53_ssu_r207_Genus.fa.gz) [40].

The decontam package (version 1.20.0) [41] was used to remove contaminating sequences by comparing the dataset (64 samples) with the two negative control samples. The function isContaminant used the following arguments: method = “prevalence”, neg = “is.neg”, threshold = 0.45. Initial quality investigation of the sequences revealed that sample E227_D_2 may have been contaminated, as its ASV composition was vastly different than all other samples and contained groups of bacteria widely known as contaminants in this type of analysis (for example, *Eubacterium*, *Faecalibacterium*, *Lactobacillus*, *Pseudomonas* [42]), and was removed from the dataset (leaving 63 samples). Decontam was then run again on the remaining 63 samples with the same settings.

### 2.5. Core Community

To identify the core microbial community of the sediments, the microbiome package (version 1.24.0) [43] was used. With the core_members-function, the core community was defined by including ASVs with abundance >0 in ≥90% of the samples (≥57 samples). The ASVs not qualified for the core group are referred to as the “non-core”, i.e., the variable community.

### 2.6. Beta-Diversity

Relative abundances of the ASV’s agglomerated to phylum level were calculated using the phyloseq R-package (version 1.46.0) [44] on the whole dataset. The top 10 abundant phyla (based on the mean relative abundance across the samples) were selected for visualization, and all other phyla were grouped together as “Other”. The ggplot2 package (version 3.4.2) [45] was used for visualization.

For beta-diversity analyses, the ASV-table was first normalized using the function rarefy_even_depth from the phyloseq package. The function vegdist of the vegan package (version 2.6-4) [46] was then used for estimating beta-diversity with the presence-absence Jaccard similarity index, followed by the construction of principal coordinate analysis (PCoA) plots using the function cmdscale. The Jaccard similarity index was also used for permutational multivariate analysis of variance (PERMANOVA) [47] analysis using function adonis2 from vegan. Pairwise comparisons with Benjamini–Hochberg correction were carried out using pairwiseAdonis-package (version 0.4.1) [48].

### 2.7. Impact of Sewage Taxa

A curated source tracking approach was conducted in the same manner as Frank et al., 2024 [9], although in this study, 38 genera were used based on a literature survey of taxa found in sewage and gut. The ggplot2 package was used for visualization.A complete list of taxa and respective references is shown in Table S2. Visualization of both the abundance of *E. coli* and the sewage taxa over the sampling area and over time is presented using GIS (QGIS version 3.28.12-Firenze).

Both Pearson correlation and Spearman’s rank correlation (function cor.test) were utilized to explore connections between continuous variables in the datasets. This includes the relative abundance of sewage taxa, with distance to WWO. When grouping samples into two groups, the Wilcoxon rank sum test was utilized. Package coda4microbiome (version 0.2.2) [49] was used to assess which taxa can be linked (either positively or negatively) to continuous variables. Only the sequences defined as non-core were included, and all samples were included. The abundances of each taxon were summed together and sorted, and the 500 taxa with the highest total abundance were extracted into a new data frame. The function coda_glmnet was used to analyze the sewage taxa relative abundance.

## 3. Results

### 3.1. Core and Non-Core Microbial Community in the Coastal Sediments

**Figure 1:**
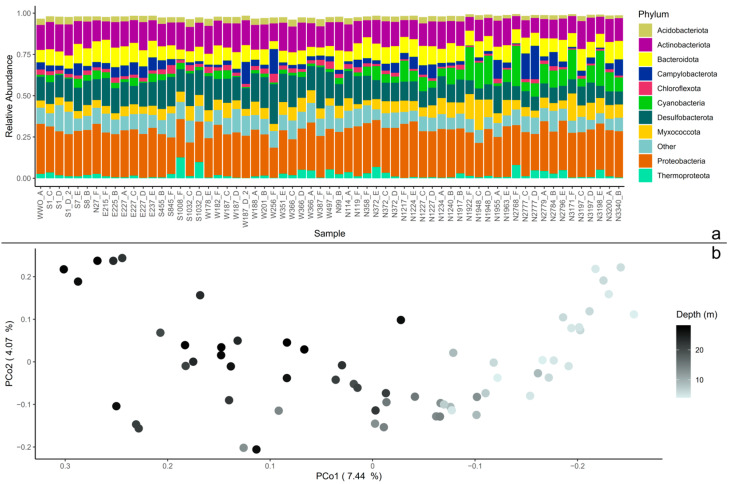
Beta diversity in the microbial community. (**a**) The whole dataset agglomerated to phylum level, displayed as relative abundance. The 10 most abundant phyla are displayed, and the rest of the remaining phyla are grouped together as “Other”. The samples (*x*-axis) are sorted by the distance from the wastewater outlet (WWO). Relative abundances of amplicon sequence variants (ASVs) were calculated so that each sample’s total abundance was normalized to 1. (**b**) Principal coordinates analysis (PCoA) plot of the whole sediment community. The axis’ eigenvalues are expressed in proportion to the sum of all eigenvalues (in percentage).

The 16s rRNA V3-V4 sequence dataset (63 samples) of coastal sediments contained 95,277 ASVs. When agglomerated at the phylum level and sorted with distance from the WWO, the relative abundance of Cyanobacteria increased with distance to WWO, which were also shallower sediment samples. Other abundant phyla present in the sediment samples were Proteobacteria (Pseudomonadota), Actinobacteriota (Actinomycetota), Desulfobacteriota, and Bacteroidota (Figure 1a).

The core community consisted of 260 ASVs, and when subtracting these ASVs from the whole dataset, 95,017 ASVs were identified and hereafter referred to as the non-core. The core community of 260 ASVs represented, on average, 38 ± 4.5% (mean ± standard deviation) of the total abundance of each sample.

The sediment core community was foremostly represented (in terms of abundance) by the genera JADHO01 (15 ± 7.8%) (in the order Polyangiales in the Myxococcota phylum), DRQR01 (8.4 ± 2.3%) (in the class Acidimicrobiia in the phylum Actinobacteria), *Sulfurovum* (6.9 ± 6.6%) (in the order Campylobacterales), S5133MH16 (5.6 ± 1.7%) (in the order Desulfobacterales), Ilumatobacter_A (5.0 ± 1.6%) (in the class Acidimicrobiia in the phylum Actinobacteria), *Nitrosopumilus* (3.5 ± 3.9%) (an archaea in the phylum Thermoproteota) and *Parahaliea* (3.1 ± 1.2%) (in the family Halieaceae within the class Gammaproteobacteria). At the phylum level, the most abundant phyla in the core were Proteobacteria (24 ± 3.6%), Actinobacteria (23 ± 3.4%), Desulfobacterota (17 ± 4.4%), Myxococcota (15 ± 7.7%) and Campylobacterota (7.3 ± 6.8%).

To visualize patterns in the presence–absence of ASVs, for the whole dataset, the core, and the non-core communities, the Jaccard similarity index was used on a rarefied ASV-table to construct PCoA and conduct PERMANOVA. For the whole dataset, the first dimension of the PCoA shows that 7.44% of the microbial community shifts over the depth profile of the samples (Figure 1b). This was also the case for the non-core dataset (Figure S1a), while in the core community, most samples were grouped together (Figure S1b). Indeed, the variance in the whole dataset and non-core could be explained by sediment depth (PERMANOVA, *p* = 0.001 for both), but also with distance to WWO (*p* = 0.001 for both), and cardinal direction in reference to WWO (*p* = 0.035 for whole dataset and *p* = 0.039 for non-core). For the core group, the only parameter with significance was depth (*p* = 0.001). However, as sediment depth and distance to WWO were linked, as the samples closest to WWO were the deepest ones, it was difficult to untangle the individual effects of depth and distance. To look into which cardinal directions were important for the results, pairwise comparisons with Benjamini–Hochberg *p*-value adjustment were conducted, and it revealed that the only pairs that are not significantly different were East-WWO and South-West (whole dataset and non-core, *p* > 0.05).

### 3.2. Viable E. coli

**Figure 2:**
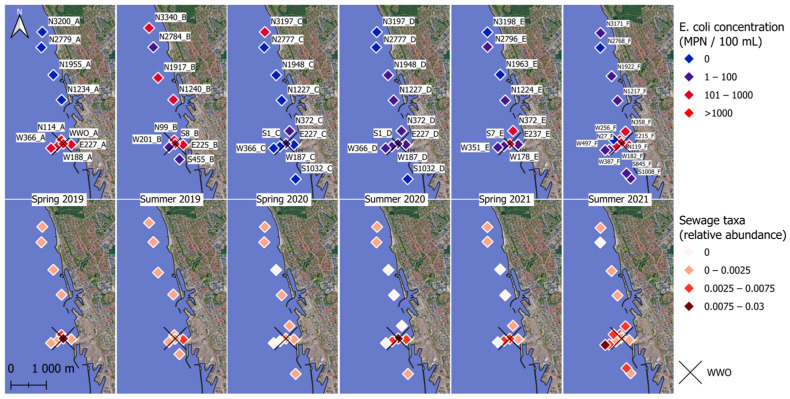
Sampling points and *E. coli* concentration and relative abundance of sewage taxa. The top row shows the *E. coli* numbers in color gradient, and the bottom row shows the relative abundance of sewage taxa in color gradient. 0.003 indicates 0.3% of a sample’s total abundance. The X marks the spot for the city’s treated wastewater outlet (WWO) point. Sample names are displayed in the top row.

Viable *E. coli* concentrations in the sediments were between 0 and 2793 MPN/100 mL, with a mean of 148 MPN/100 mL and a median of 24 MPN/100 mL. In spring 2019, spring 2021, and summer 2021, the highest *E. coli* concentrations tended to occur near WWO. Viable *E. coli* >100 MPN/100 mL were observed occasionally at sample sites more than one kilometer away from the WWO, and, on two occasions, were observed up to three kilometers north of the WWO (Figure 2).

### 3.3. Impact of Sewage Taxa

**Figure 3:**
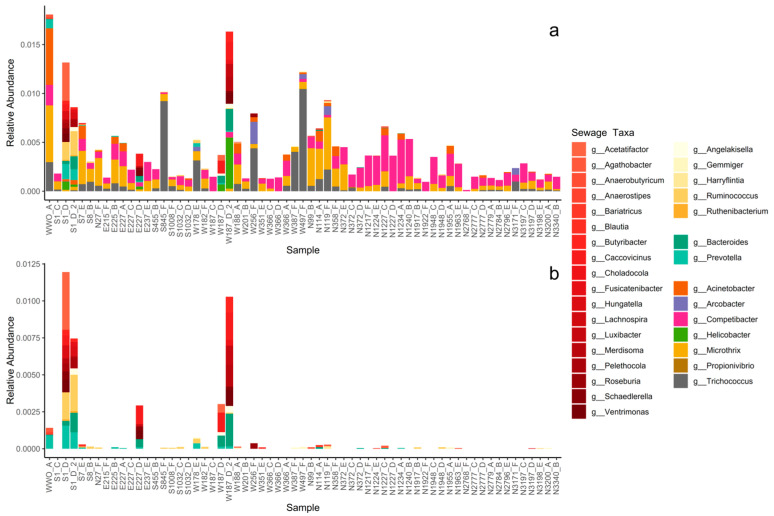
Curated source tracking analysis. (**a**) Relative abundance of each sewage taxa for each sediment sample (sorted by distance to WWO). Abundances are presented at genus level with family implied by color grouping: taxa in the family Lachnospiraceae are colored in reds, taxa in the family Ruminococcaceae are colored in yellows, taxa in the family Bacteroidaceae are colored in mint greens. The remaining seven taxa are all from different families and thus colored individually. (**b**) Relative abundance of only the families Lachnospiraceae, Ruminococcaceae, and Bacteroidaceae; the same colors as in Figure 3a are used.

Thirty-eight genera related to microbial communities found in sewage or the gut were present in the sediments (Table S2) [50,51,52,53,54,55,56,57,58,59,60,61,62,63,64,65,66,67,68,69,70,71,72,73]. When summed together, sewage taxa represented less than 1% of the total sediment community but did occur in all sediment samples (Figure 2 and Figure 3a). None of the sewage taxa were part of the core microbial community, showing an uneven spatiotemporal distribution of the individual sewage taxa, i.e., individual taxa were restricted to a few sampling locations or times.

The highest abundance of sewage taxa tended to occur near WWO (Figure 2 and Figure 3a), and indeed a moderate correlation was observed with distance to WWO (63 samples) (Pearson *p*-value < 0.01, cor = −0.41; Spearman *p*-value < 0.01, rho = −0.46). When looking at seasonality, the median relative abundance is not significantly different between spring and summer (Wilcoxon rank sum test, *n* = 28 + 35, *p*-value = 0.93). A relatively high abundance of sewage taxa was also noticed around 1.2 km north of WWO in a region of sandy sediment (Figure 2 and Figure 3).

The sewage taxa with the highest abundance in the sediments were Competibacter_A, *Microthrix*, and *Trichococcus*, which are bacteria commonly observed in WWTPs bioreactors and effluents (Table S2), suggesting perhaps an origin from the treated effluent of the local WWTP. For the sediment sample closest to the WWTP effluent, WWO_A, a high abundance of *Acinetobacter* was noticed.

For other sewage taxa, localized short-lived spikes in relative abundance were observed (Figure 3a). To determine if some of these taxa might originate from untreated sewage sources, such as CSO, the presence of bacterial groups that are highly abundant in the human gut was investigated for the sediment samples. The taxa selected as “gut taxa” were genera in the families Lachnospiraceae [74], Ruminococcaceae [75], and Bacteroidaceae [62]. These were observed in samples E227_D, S1_D, S1_D_2, W187_D, and W187_D_2 (Figure 3b), all taken during the summer of 2020, and all located in the same geographical area, south of a known CSO outlet. Locations for the CSO outlet and stormwater outlet are not displayed in Figure 2 due to confidentiality. The occurrence of CSO discharges for up to three months prior to sampling (confidential data) was investigated for all six sampling times, revealing that a CSO event happened during the summer of 2020. Interestingly, the relative abundance of these taxa was much lower for the same locations in the spring of 2021 samples, suggesting that the effects of CSO discharges in sediments are temporary.

**Figure 4:**
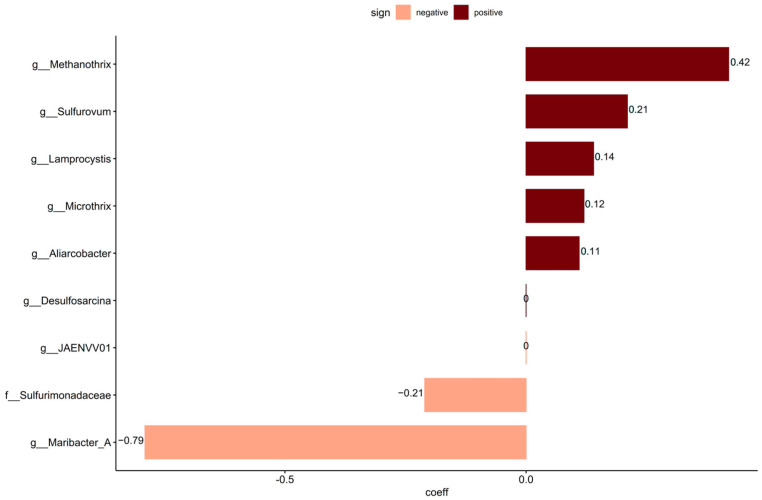
Penalized linear regression analysis on continuous data using the relative abundance of sewage taxa on the non-core data identified taxa with positive values (dark red) and taxa with negative values (peach) connected to higher sewage taxa presence.

To identify taxa that may not have been included in the curated source tracking, coda4microbiome was used to link continuous values of sewage taxa to the non-core community. Analyzing these taxa for the penalized linear regression analysis resulted in eight significant new taxa that were not included in the curated source tracking taxa set. Of the nine significant taxa detected, six had positive and three had negative log-contrast coefficients to the relative abundance of sewage taxa data (Figure 4). The non-core taxa positively linked with sewage taxa were the genera *Methanotrix*, *Sulfurovum*, *Lamprocystis*, *Microthrix*, and *Aliarcobacter*.

## 4. Discussion

The microbial communities in the studied urban coastal sediments were similar to other oxic marine sediments, where Thermoproteota (Thaumarchaeota) and Proteobacteria often dominate [76]. However, the frequent detection of *E. coli* and other sewage- and gut-associated taxa indicates anthropogenic contamination in this area.

The likely nearby point sources of *E. coli* and sewage taxa in sediments are suggested to be the effluent of the wastewater treatment plant (corresponding to WWO), as well as a CSO effluent north of WWO. Sediments closest to the WWO consistently harbored viable *E. coli* present and generally exhibited higher proportions of sewage taxa, suggesting the survival of bacteria in treated wastewater, as previously reported in Anastasi, et al. [77], Aslan, et al. [78], Raboni, et al. [79].

Sediments can serve as a reservoir for *E. coli* with the aid of biofilm formation [15,80], meaning that *E. coli* concentrations observed may be due to the local survival of viable cells [81,82,83]. The data in the present study indicate at least short-term survival in the marine environment, also verified by Byrd and Colwell [84], who observed persistence for up to three years.

Viable *E. coli* and sewage-associated taxa were observed in sediments between 3171–3340 m north of the WWO, suggesting that these bacteria originate from neither the WWO effluent nor CSO. The detection of potential extraintestinal pathogenic *E. coli* phylogroup B2 [85] in the northernmost sediments also supports the presence of other possible sources of contamination [29]. Potential sources include stormwater, a known source for *E. coli* [86], fecal contamination [87], and naturalization of *E. coli.* Survival of *E. coli* has been shown in multiple environments, such as water [88], beach sand [89,90], soil [91], and sediments [92,93]. Naturalized *E. coli* can survive and reproduce independently of recent contamination events [94], challenging the usefulness and reliability of *E. coli* as a FIB.

The localized presence of gut bacteria during one of the sampling times could indicate untreated sewage discharges, which is likely associated with a recent CSO event. Lachnospiraceae and Ruminococcaceae are highly abundant in influent sewage (not yet treated sewage entering the WWTP) [95,96], and their presence in these samples supports an impact on the sediment community by the CSO. The CSO outlet is north of all these four locations, suggesting a southward direction of the flow, which is consistent with the usual southward flow of high salinity water below the halocline [31,97]. Even though the CSO volume is low (≤0.55%) [98] in relation to the overall discharge to the area (i.e., WWO and CSO combined), its influence on FIB levels in coastal areas should not be disregarded [99]. Additionally, other intermittent sources of fecal contamination might be influencing the sediments of the area, such as boat traffic [100], animals [101,102], and rainfall [103].

*Arcobacter* is largely abundant in sewage (both influent and effluent) [59], and was found exclusively in sediments containing a high abundance of *Trichococcus*, which are also known to be highly abundant in sewage influent and effluent [59]. Stormwater effluent has been indicated as a potential source of *Arcobacter* [104], indicating stormwater’s contribution to sediment microbial communities. However, as the microbial community composition in stormwater varies largely both spatially and temporally, source tracking of stormwater is complex [105]. Locations distant from WWO or CSO effluent may be contaminated by stormwater, stormwater containing wastewater, or other unidentified sources.

Several taxa whose abundance was potentially linked with sewage were detected using penalized linear regression (coda4microbiome). This included *Microthrix*, which is also one of the taxa included in the curated source tracking library. *Methanothrix* is an anaerobic archaea that uses acetate as an energy source [106] and is often found in anaerobic digestors at WWTPs [107]. *Sulfurovum* was also identified and is chemolithoautotrophic, oxidizing hydrogen, elemental sulfur, and thiosulfate, and can reduce oxygen, nitrate, thiosulfate, and elemental sulfur. *Sulfurovum* requires a saline environment for growth and has been found in ocean sediments [108]. It was also identified as one of the taxa in the core community, suggesting that it is a natural inhabitant of the sediments. Its co-occurrence with sewage taxa might be due to the addition of sulfur and sulfate in the effluent from the treatment plant, indicating a potential impact of the wastewater in the natural sulfur cycling of sediments. This could also be the case for *Lamprocystis*, a photolithoautotrophic sulfur oxidizer bacteria [109], and *Desulfosarcina*, a sulfate reducer [110]. The detection of sequences from *Aliarcobacter* was a cause for concern as this is a human pathogen causing enteritis, septicemia, and bacteremia and is found in infected human feces. Due to its survivability in various water environments such as wastewater, seawater, and freshwater [111], the presence of live *Aliarcobacter* in coastal sediment would pose a risk of human exposure and infection. *Aliarcobacter* has also been observed in sewage and CSO discharges [27]. Resuspension and transport of the reservoir matter over the bed surface may result in intermittent contamination of coastal bathing water and could be a public hazard. Further investigations should determine if these bacteria are alive, as DNA sequencing-based investigations are not able to assess this and, thus, can only be used as indications of risk to trigger additional investigation.

The correlation between sewage taxa abundance and distance to the WWO indicates that treated effluent is a significant contamination source. Due to fluctuations in abundance and irregular patterns in spread through the sediments, an exact pattern in the sewage taxa distribution was not evident, likely compounded by the complication that not all effluent sources could be identified and addressed in this study. Furthermore, the sampling interval of six months does limit the resolution of microbial community and *E. coli* data such that pinpointing pollution events from intermittent sources (CSOs, stormwater) is challenging. In order to fully conclude the effects from these diffuse sources, future studies could benefit from performing event-driven sampling, such as before, during and after large rainfall. Considering that the geographical position of the Öresund strait includes an international border, the determination of the effluent source can be a challenging task [112,113]. Ultimately, further research is necessary to confirm the effluent origins and transport conditions, including investigation of flow directions, plume propagation and diffusion, the impact of coastal processes, and meteorological conditions.

As with all methods, it is important to consider their inherent inaccuracies and limitations. The 16S rRNA gene dataset is a DNA-based method, which cannot distinguish alive or dead bacteria at the time of sampling. In addition to this, high variation in the taxonomic composition between pairs of duplicate samples may indicate a high spatial variation of taxa in the sediments. Some of the taxa used for source tracking in this study may also unknowingly occur in the environment, which adds uncertainties to the identification of sources of contamination. That might be the case for *Microthrix*, where a marine clade exists [114], while for *Competibacter*, their ecology outside WWTP bioreactors has not been well explored. Even with analyses based on the enzymatic activity of viable *E. coli*, there are challenges. *E. coli* can enter a viable but nonculturable (VBNC) state in ocean water [115], and then it would not be detected and counted in the Colilert18 analysis. Not least, it is important to consider the sample representability in relation to the heterogeneous mass of the corresponding sediment deposit [116]. While this study’s focus lies on the surface layer of sediments to investigate recent pollution events, it is important to take into consideration that due to sediment mixing, lower layers of sediments can resurface. This, in combination with the naturally high spatial variability and diversity of microorganisms in sediments, causes high complexity in analyses. While top layer sediments also have been found important in other studies [117], to address these complexities, it could be fruitful to investigate deeper down in the sediment core, as well as explore different core diameter sizes.

## 5. Conclusions

The present study examined microbial communities in urban coastal sediments that may have been impacted by different sources of contamination and included viable *E. coli*, over three years. Clear indications of sewage influencing the sediment microbial community were observed. However, no significant seasonal variation in *E. coli* or sewage and gut taxa was found between spring and summer. A wastewater treatment plant (WWTP) and combined sewer overflow (CSO) were contributors of *E. coli* and other sewage-associated taxa to the sediments, though higher concentrations at locations located further away from the CSO and WWTP outlets considered in this study implied other sources, including potentially stormwater.

The results from this study add support to sediments acting as long-term reservoirs for *E. coli*. The study identified certain microbial taxa (e.g., Lachnospiraceae, *Prevotella*, *Bacteroides*, *Trichococcus*, and *Arcobacter*) linked to specific sewage sources, showing how traditional FIB surveys can benefit from the addition of 16S rRNA gene sequencing.

Further research is needed to confirm contamination origins, including the possible role of stormwater, determine the importance of *E. coli* naturalization, and assess possible public health risks.

## Data Availability

The raw sequencing data generated in this study have been deposited in the NCBI SRA database under accession code PRJNA1116073.

## Supplementary Materials

The following supporting information can be downloaded at: https://www.mdpi.com/article/10.3390/microorganisms13050983/s1, Figure S1. (a) Principal coordinates analysis (PCoA) plot of non-core sediment community. The axis’ eigenvalues are expressed in proportion to the sum of all eigenvalues (in percentage); (b) Principal coordinates analysis (PCoA) plot of core sediment community. The axis’ eigenvalues are expressed in proportion to the sum of all eigenvalues (in percentage); Table S1. Sampling metadata for all sediments collected; Table S2. The 38 taxa/genera used in the curated source tracking analysis, as well as information regarding where each taxon previously has been found and which references support this claim.
