# Supplementary material for: Microbial Contamination in Urban Marine Sediments: Source Identification Using Microbial Community Analysis and Fecal Indicator Bacteria
Source: Microorganisms. 2025 Apr 25;13(5):983. doi: 10.3390/microorganisms13050983 (PMC12114436; doi:10.3390/microorganisms13050983)
Supplement: Supplementary file 1 [file microorganisms-13-00983-s001.zip › microorganisms-3576203-supplementary.pdf]

Supplementary Materials

Table S1. Sampling metadata for all sediments collected.

Sample	GPS-DD Latitude	GPS-DD Longitude	Distance (m)	Depth (m)	*E. coli* concentration (MPN/100mL)	Season	Year
E215_F	56,03252	12,68777	214.89	13.9	163	Summer	2021
E225_B	56,03265	12,68797	224.59	13.7	322	Summer	2019
E227_A	56,03273	12,68802	226.69	13	531	Spring	2019
E227_C	56,03271	12,68803	227.44	13	0	Spring	2020
E227_D	56,03271	12,68803	227.44	13	21	Summer	2020
E237_E	56,03272	12,68818	236.91	13	28	Spring	2021
N114_A	56,03382	12,6835	114.12	21	2793	Spring	2019
N119_F	56,03388	12,68355	119.23	20.2	156	Summer	2021
N1217_F	56,0438	12,68223	1217.48	7.2	34	Summer	2021
N1224_E	56,04385	12,6821	1223.94	9	32	Spring	2021
N1227_C	56,04389	12,68225	1227.01	9	0	Spring	2020
N1227_D	56,04389	12,68225	1227.01	9	73	Summer	2020
N1234_A	56,044	12,68338	1233.94	4	0	Spring	2019
N1240_B	56,04405	12,68327	1239.87	4.9	104	Summer	2019
N1917_B	56,04955	12,67625	1917.25	8.9	126	Summer	2019
N1922_F	56,04998	12,67943	1922.4	5.9	8	Summer	2021
N1948_C	56,05023	12,6796	1948.09	4	0	Spring	2020
N1948_D	56,05023	12,6796	1948.09	4	5	Summer	2020
N1955_A	56,05028	12,67952	1954.52	7	0	Spring	2019
N1963_E	56,05037	12,67968	1962.55	5	0	Spring	2021
N27_F	56,0331	12,6841	26.89	19.7	280	Summer	2021
N2768_F	56,05715	12,67418	2768.05	5.6	0	Summer	2021
N2777_C	56,05722	12,67405	2777.11	6	0	Spring	2020
N2777_D	56,05722	12,67405	2777.11	6	0	Summer	2020
N2779_A	56,05722	12,67395	2778.64	6	0	Spring	2019
N2784_B	56,05727	12,67397	2783.8	6	20	Summer	2019
N2796_E	56,05735	12,67377	2795.64	6	3	Spring	2021
N3171_F	56,06087	12,67422	3171.38	4.6	5	Summer	2021
N3197_C	56,06111	12,67426	3197.4	4.8	154	Spring	2020
N3197_D	56,06111	12,67426	3197.4	4.8	0	Summer	2020
N3198_E	56,06105	12,67367	3198.21	4.5	0	Spring	2021
N3200_A	56,06113	12,67427	3199.76	5	0	Spring	2019
N3340_B	56,06213	12,67192	3339.64	4.8	172	Summer	2019
N358_F	56,036	12,68603	357.81	14.4	111	Summer	2021
N372_C	56,03614	12,68597	372.05	15.3	88	Spring	2020
N372_D	56,03614	12,68597	372.05	15.3	42	Summer	2020
N372_E	56,03617	12,68578	371.98	14.5	260	Spring	2021
N99_B	56,03377	12,68393	98.56	21	150	Summer	2019
S1_C	56,03291	12,6844	1.45	21.4	30	Spring	2020
S1_D	56,03291	12,6844	1.45	21.4	92	Summer	2020
S1_D_2	56,03291	12,6844	1.45	21.4	92	Summer	2020
S1008_F	56,02417	12,6886	1007.62	23.3	31	Summer	2021

S1032_C	56,024	12,68899	1031.82	23.7	0	Spring	2020
S1032_D	56,024	12,68899	1031.82	23.7	0	Summer	2020
S455_B	56,02898	12,68638	454.75	22.7	62	Summer	2019
S7_E	56,03285	12,68437	7.49	22	160	Spring	2021
S8_B	56,03285	12,68433	8.07	21.3	350	Summer	2019
S845_F	56,02543	12,68672	844.72	23.4	24	Summer	2021
W178_E	56,03232	12,68172	178.27	26.5	5	Spring	2021
W182_F	56,03218	12,68177	181.87	25.9	454	Summer	2021
W187_C	56,03222	12,68165	186.82	26.5	0	Spring	2020
W187_D	56,03222	12,68165	186.82	26.5	13	Summer	2020
W187_D_2	56,03222	12,68165	186.82	26.5	13	Summer	2020
W188_A	56,03222	12,68163	187.74	26	431	Spring	2019
W201_B	56,03202	12,68158	200.69	27.1	20	Summer	2019
W256_F	56,03393	12,68068	256.13	22.4	0	Summer	2021
W351_E	56,03195	12,679	351.29	28.2	8	Spring	2021
W366_A	56,03175	12,67887	366.44	28	385	Spring	2019
W366_C	56,03174	12,67889	365.76	28.2	0	Spring	2020
W366_D	56,03174	12,67889	365.76	28.2	3	Summer	2020
W387_F	56,03138	12,6788	386.53	27.5	15	Summer	2021
W497_F	56,03125	12,67697	496.63	27.9	24	Summer	2021
WWO_A	56,03292	12,68438	0	22	1240	Spring	2019

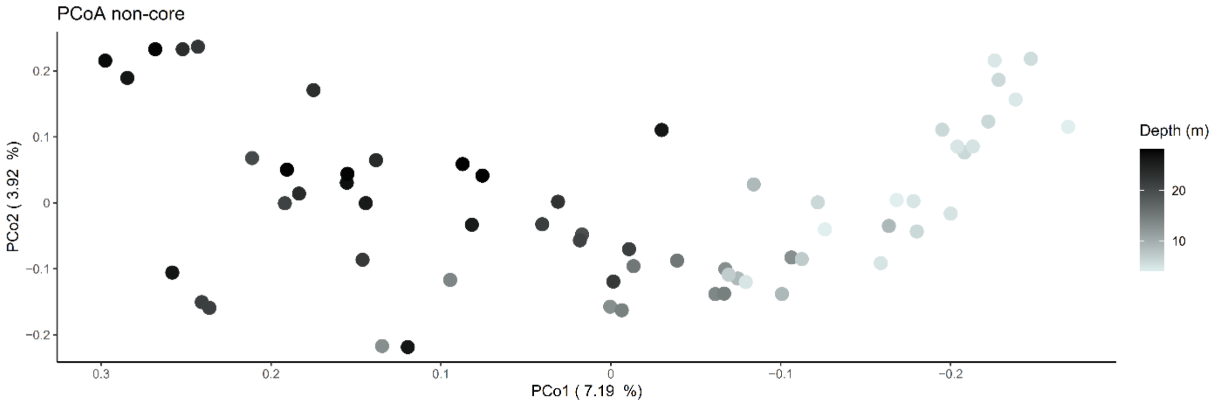

Figure S1. (a) Principal coordinates analysis (PCoA) plot of non-core sediment community. The axis' eigenvalues are expressed in proportion to the sum of all eigenvalues (in percentage).

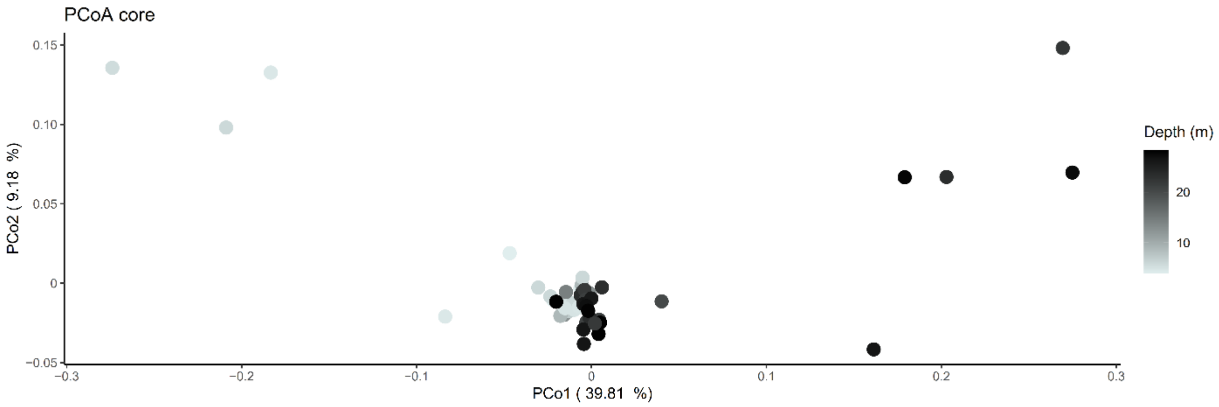

Figure S1. (b) Principal coordinates analysis (PCoA) plot of core sediment community. The axis' eigenvalues are expressed in proportion to the sum of all eigenvalues (in percentage).

Table S2. The 38 taxa/genera used in the curated source tracking analysis, as well as information regarding where each taxon previously has been found and which references support this claim.

Sewage and gut-related taxonomic group	Sample and geographic region	Reference
Genus *Acetatifactor*	Caecum (intestines) of mice, Germany	https://doi.org/10.1007/s00203-012-0822-1 (Pfeiffer et al., 2012)
	Fecal, adult hens, England	https://doi.org/10.7717/peerj.10941 (*Caccovicinus*, *Choladocola*, *Merdisoma*, *Pelethocola*, *Ventrimonas* also reported) (Gilroy et al., 2021)
Genus *Acinetobacter*	Sewage influent & fecal, adult USA	https://doi.org/10.1111/j.1462-2920.2009.02075.x (*Bacteroides*, *Arcobacter* & *Trichococcus* (only in sewage) also reported) (McLellan et al., 2010)
	In the established global core (OTUs) in	https://doi.org/10.1038/s41564-019-0426-5 (*Arcobacter* also reported) (Wu et al., 2019)

	activated sludge.	
	Global study of activated sludge (ASVs)	https://doi.org/10.1038/s41467-022-29438-7 (*Arcobacter*, *Ca. Competibacter*, *Ca. Microthrix*, *Prevotella*, *Propionivibrio*, *Trichococcus* also reported) (Dueholm et al., 2022)
Genus *Agathobacter*	Fecal, 2.5 year olds, China	https://doi.org/10.1186/s12941-022-00535-1 (*Bacteroides*, *Blautia*, *Hungatella*, *Lachnospira*, *Roseburia* also reported) (Li et al., 2022)
Genus *Anaerobutyricum*	Fecal, infant, United Kingdom	https://doi.org/10.1099/ijsem.0.003041 (Shetty et al., 2018)
Genus *Anaerostipes*	Fecal, adult, United Kingdom	https://doi.org/10.1078/0723-2020-00096 (Schwiertz et al., 2002)
Genus *Angelakisella*	Fecal, mice, USA	https://doi.org/10.1038/s41366-020-00712-2 (*Anaerostipes*, *Bacteroides*, *Harryflintia*, *Roseburia*, *Ruminococcus* also reported) (Qiu et al., 2020)
Genus *Arcobacter*	Sewage influent,	https://doi.org/10.1128/AEM.03044-19

	process tank & sewage effluent Denmark	(*Bacteroides, Blautia, Ca. Competibacter, Ca. Microthrix, Trichococcus* also reported) (Kristensen et al., 2020)
	Sewage influent, USA	https://doi.org/10.1111/j.1462-2920.2009.02075.x (*Acinetobacter*, *Bacteroides*, *Trichococcus* also reported) (McLellan et al., 2010)
	In the established global core (OTUs) in activated sludge.	https://doi.org/10.1038/s41564-019-0426-5 (*Acinetobacter* also reported) (Wu et al., 2019)
	Global study of activated sludge (ASVs)	https://doi.org/10.1038/s41467-022-29438-7 (*Acinetobacter*, *Ca. Competibacter*, *Ca. Microthrix*, *Prevotella*, *Propionivibrio*, *Trichococcus* also reported) (Dueholm et al., 2022)
Genus *Bacteroides* (g__Bacteroides and	Fecal, adult	https://doi.org/10.1038/nature11053 (*Helicobacter*, *Prevotella*, *Roseburia* also reported)

g__Bacteroides_G in GTDB)	Venezuela, Malawi, USA	(Yatsunenko et al., 2012)
	Sewage influent & fecal, adult USA	https://doi.org/10.1111/j.1462-2920.2009.02075.x (*Acinetobacter*, *Arcobacter* & *Trichococcus* (only in sewage) also reported) (McLellan et al., 2010)
	Fecal, adult, Denmark, Spain	https://doi.org/10.1038/nature08821 (*Blautia*, *Roseburia*, *Ruminococcus* also reported) (Qin et al., 2010)
	Sewage influent, process tank & sewage effluent Denmark	https://doi.org/10.1128/AEM.03044-19 (*Arcobacter*, *Blautia*, *Ca. Competibacter*, *Ca. Microthrix*, *Trichococcus* also reported) (Kristensen et al., 2020)
	Fecal, adult, USA	https://doi.org/10.1038/nature11234 (*Helicobacter*, *Prevotella* also reported) (Huttenhower et al., 2012)
Genus *Bariatricus*	Fecal, adult, China, USA	https://doi.org/10.1038/s41467-022-32991-w

		(*Acetatifactor*, *Agathobacter*, *Anaerostipes*, *Bacteroides*, *Blautia*, *Gemmiger*, *Hungatella*, *Roseburia*, *Ruminococcus* also reported)<br><br>(Ke et al., 2022)
Genus *Blautia*<br>(g__Blautia_A in GTDB)	Fecal, adult, China, USA	https://doi.org/10.1038/s41467-022-32991-w<br><br>(*Acetatifactor*, *Agathobacter*, *Anaerostipes*, *Bacteroides*, *Bariatricus*, *Gemmiger*, *Hungatella*, *Roseburia*, *Ruminococcus* also reported)<br><br>(Ke et al., 2022)
	Fecal, adult, Denmark, Spain	https://doi.org/10.1038/nature08821<br>(*Bacteroides*, *Roseburia*, *Ruminococcus* also reported)<br>(Qin et al., 2010)
	Sewage influent, process tank & sewage effluent Denmark	https://doi.org/10.1128/AEM.03044-19<br>(*Arcobacter*, *Bacteroides*, *Ca. Competibacter*, *Ca. Microthrix*, *Trichococcus* also reported)<br>(Kristensen et al., 2020)
Genus *Butyribacter*	Fecal, adult, China	https://doi.org/10.1016/j.syapm.2021.126201<br>(Zou et al., 2021)

Genus *Caccovicinus*	Fecal, adult hens, England	https://doi.org/10.7717/peerj.10941 (*Acetatifactor*, *Choladocola*, *Merdisoma*, *Pelethocola*, *Ventrimonas* also reported) (Gilroy et al., 2021)
Genus *Ca. Competibacter* (g__Competibacter and g__Competibacter_A in GTDB)	Activated sludge, Denmark	https://doi.org/10.1038/ismej.2013.162 (McIlroy et al., 2014)
	Sewage influent, process tank & sewage effluent Denmark	https://doi.org/10.1128/AEM.03044-19 (*Arcobacter*, *Bacteroides*, *Blautia*, *Ca. Microthrix*, *Trichococcus* also reported) (Kristensen et al., 2020)
	Global study of activated sludge (ASVs)	https://doi.org/10.1038/s41467-022-29438-7 (*Acinetobacter*, *Arcobacter*, *Ca. Microthrix*, *Prevotella*, *Propionivibrio*, *Trichococcus* also reported) (Dueholm et al., 2022)
Genus *Ca. Microthrix* (g__Microthrix in GTDB)	Activated sludge Denmark	https://doi.org/10.1016/j.watres.2020.115955 (*Trichococcus* also reported) (Nierychlo et al., 2020)
	Sewage influent,	https://doi.org/10.1128/AEM.03044-19

	process tank & sewage effluent Denmark	(*Arcobacter*, *Bacteroides*, *Blautia*, *Ca. Competibacter*, *Trichococcus* also reported) (Kristensen et al., 2020)
	Global study of activated sludge (ASVs)	https://doi.org/10.1038/s41467-022-29438-7 (*Acinetobacter*, *Arcobacter*, *Ca. Competibacter*, *Prevotella*, *Propionivibrio*, *Trichococcus* also reported) (Dueholm et al., 2022)
Genus *Choladocola*	Fecal, adult hens, England	https://doi.org/10.7717/peerj.10941 (*Acetatifactor*, *Caccovicinus*, *Merdisoma*, *Pelethocola*, *Ventrimonas* also reported) (Gilroy et al., 2021)
Genus *Fusicatenibacter*	Fecal, adult, Japan	https://doi.org/10.1099/ijs.0.045823-0 (Takada et al., 2013)
Genus *Gemmiger*	Fecal, adult, China, USA	https://doi.org/10.1038/s41467-022-32991-w (*Acetatifactor*, *Agathobacter*, *Anaerostipes*, *Bacteroides*, *Bariatricus*, *Blautia*, *Hungatella*, *Roseburia*, *Ruminococcus* also reported) (Ke et al., 2022)

Genus *Harryflintia*	Caecum (intestines) of chicken, Germany	https://doi.org/10.1099/ijsem.0.001317<br>(Petzoldt et al., 2016)
Genus *Helicobacter* (g__Helicobacter_C, g__Helicobacter_D and g__Helicobacter_I in GTDB)	Sewage influent Mexico	https://doi.org/10.1128/AEM.68.3.1436-1439.2002<br>(Lu et al., 2002)
	Fecal, adult Venezuela, Malawi, USA	https://doi.org/10.1038/nature11053<br>(*Bacteroides*, *Prevotella*, *Roseburia* also reported)<br>(Yatsunenko et al., 2012)
	Fecal, adult, USA	https://doi.org/10.1038/nature11234<br>(*Bacteroides*, *Prevotella* also reported)<br>(Huttenhower et al., 2012)
Genus *Hungatella* (g__Hungatella_A in GTDB)	Fecal, adult, China, USA	https://doi.org/10.1038/s41467-022-32991-w<br>(*Acetatifactor*, *Agathobacter*, *Anaerostipes*, *Bacteroides*, *Bariatricus*, *Blautia*, *Gemminger*, *Roseburia*, *Ruminococcus* also reported)<br>(Ke et al., 2022)

Genus *Lachnospira*	Fecal, 2.5 year olds, China	https://doi.org/10.1186/s12941-022-00535-1 (*Agathobacter*, *Bacteroides*, *Blautia*, *Hungatella*, *Roseburia* also reported) (Li et al., 2022)
Genus *Luxibacter*	Fecal, adult, France	https://doi.org/10.1016/j.nmni.2021.100850 (Naud et al., 2021)
Genus *Merdisoma*	Fecal, adult hens, England	https://doi.org/10.7717/peerj.10941 (*Acetatifactor*, *Caccovicinus*, *Choladocola*, *Pelethocola*, *Ventrimonas* also reported) (Gilroy et al., 2021)
Genus *Pelethocola*	Fecal, adult hens, England	https://doi.org/10.7717/peerj.10941 (*Acetatifactor*, *Caccovicinus*, *Choladocola*, *Merdisoma*, *Ventrimonas* also reported) (Gilroy et al., 2021)
Genus *Prevotella*	Fecal, adult Venezuela, Malawi, USA	https://doi.org/10.1038/nature11053 (*Bacteroides*, *Helicobacter*, *Roseburia* also reported) (Yatsunenko et al., 2012)
	Global study of activated sludge (ASVs)	https://doi.org/10.1038/s41467-022-29438-7 (*Acinetobacter*, *Arcobacter*, *Ca. Competibacter*, *Ca. Microthrix*, *Propionivibrio*, *Trichococcus* also reported) (Dueholm et al., 2022)

	Fecal, adult, USA	https://doi.org/10.1038/nature11234 (*Bacteroides*, *Helicobacter* also reported) (Huttenhower et al., 2012)
Genus *Propionivibrio*	Activated sludge, Denmark	https://doi.org/10.3389/fmicb.2016.01033 (Albertsen et al., 2016)
	Global study of activated sludge (ASVs)	https://doi.org/10.1038/s41467-022-29438-7 (*Acinetobacter*, *Arcobacter*, *Ca. Competibacter*, *Ca. Microthrix*, *Prevotella*, *Trichococcus* also reported) (Dueholm et al., 2022)
Genus *Roseburia*	Fecal, adult Venezuela, Malawi, USA	https://doi.org/10.1038/nature11053 (*Bacteroides*, *Helicobacter*, *Prevotella* also reported) (Yatsunenko et al., 2012)
	Fecal, adult, China, USA	https://doi.org/10.1038/s41467-022-32991-w (*Acetatifactor*, *Agathobacter*, *Anaerostipes*, *Bacteroides*, *Bariatricus*, *Blautia*, *Gemminger*, *Hungatella*, *Ruminococcus* also reported) (Ke et al., 2022)

	Fecal, 2.5 year olds, China	https://doi.org/10.1186/s12941-022-00535-1 (*Agathobacter*, *Bacteroides*, *Blautia*, *Hungatella*, *Lachnospira* also reported) (Li et al., 2022)
	Fecal, adult, Denmark, Spain	https://doi.org/10.1038/nature08821 (*Bacteroides*, *Blautia*, *Ruminococcus* also reported) (Qin et al., 2010)
	Fecal, mice, USA	https://doi.org/10.1038/s41366-020-00712-2 (*Anaerostipes*, *Angelakisella*, *Bacteroides*, *Harryflintia*, *Ruminococcus* also reported) (Qiu et al., 2020)
Genus *Ruthenibacterium*	Fecal, adult, Russia	https://doi.org/10.1099/ijsem.0.001143 (Shkoporov et al., 2016)
Genus *Ruminococcus* (g__Ruminococcus_F, g__HUN007 and g__CAG-353 in GTDB)	Fecal, adult, Denmark, Spain	https://doi.org/10.1038/nature08821 (*Bacteroides*, *Blautia*, *Roseburia* also reported) (Qin et al., 2010)
Genus *Schaedlerella*	Fecal, mice, Singapore	https://doi.org/10.1099/ijsem.0.003671 (Soh et al., 2019)
Genus *Trichococcus*	Sewage influent,	https://doi.org/10.1128/AEM.03044-19

	process tank & sewage effluent Denmark	(*Arcobacter*, *Bacteroides*, *Blautia*, *Ca. Competibacter*, *Ca. Microthrix* also reported) (Kristensen et al., 2020)
	Activated sludge Denmark	https://doi.org/10.1016/j.watres.2020.115955 (*Ca. Microthrix* also reported) (Nierychlo et al., 2020)
	Global study of activated sludge (ASVs)	https://doi.org/10.1038/s41467-022-29438-7 (*Acinetobacter*, *Arcobacter*, *Ca. Competibacter*, *Ca. Microthrix*, *Prevotella*, *Propionivibrio* also reported) (Dueholm et al., 2022)
	Sewage influent, USA	https://doi.org/10.1111/j.1462-2920.2009.02075.x (*Acinetobacter*, *Bacteroides*, *Arcobacter* also reported) (McLellan et al., 2010)
Genus Ventrimonas	Fecal, adult hens, England	https://doi.org/10.7717/peerj.10941 (*Acetatifactor*, *Caccovicinus*, *Choladocola*, *Merdisoma*, *Pelethocola* also reported) (Gilroy et al., 2021)

References

ALBERTSEN, M., MCILROY, S. J., STOKHOLM-BJERREGAARD, M., KARST, S. M. & NIELSEN, P. H. 2016. "Candidatus Propionivibrio aalborgensis": A Novel Glycogen Accumulating Organism Abundant in Full-Scale Enhanced Biological Phosphorus Removal Plants. *Front Microbiol,* 7**,** 1033.

DUEHOLM, M. K. D., NIERYCHLO, M., ANDERSEN, K. S., RUDKJØBING, V., KNUTSSON, S., ARRIAGA, S., BAKKE, R., BOON, N., BUX, F., CHRISTENSSON, M., CHUA, A. S. M., CURTIS, T. P., CYTRYN, E., ERIJMAN, L., ETCHEBEHERE, C., FATTA-KASSINOS, D., FRIGON, D., GARCIA-CHAVES, M. C., GU, A. Z., HORN, H., JENKINS, D., KREUZINGER, N., KUMARI, S., LANHAM, A., LAW, Y., LEIKNES, T., MORGENROTH, E., MUSZYŃSKI, A., PETROVSKI, S., PIJUAN, M., PILLAI, S. B., REIS, M. A. M., RONG, Q., ROSSETTI, S., SEVIOUR, R., TOOKER, N., VAINIO, P., VAN LOOSDRECHT, M., VIKRAMAN, R., WANNER, J., WEISSBRODT, D., WEN, X., ZHANG, T., NIELSEN, P. H., ALBERTSEN, M. & NIELSEN, P. H. 2022. MiDAS 4: A global catalogue of full-length 16S rRNA gene sequences and taxonomy for studies of bacterial communities in wastewater treatment plants. *Nature Communications,* 13.

GILROY, R., RAVI, A., GETINO, M., PURSLEY, I., HORTON, D. L., ALIKHAN, N.-F., BAKER, D., GHARBI, K., HALL, N., WATSON, M., ADRIAENSSENS, E. M., FOSTER-NYARKO, E., JARJU, S., SECKA, A., ANTONIO, M., OREN, A., CHAUDHURI, R. R., LA RAGIONE, R., HILDEBRAND, F. & PALLEN, M. J. 2021. Extensive microbial diversity within the chicken gut microbiome revealed by metagenomics and culture. *PeerJ,* 9.

HUTTENHOWER, C., GEVERS, D., KNIGHT, R., ABUBUCKER, S., BADGER, J. H., CHINWALLA, A. T., CREASY, H. H., EARL, A. M., FITZGERALD, M. G., FULTON, R. S., GIGLIO, M. G., HALLSWORTH-PEPIN, K., LOBOS, E. A., MADUPU, R., MAGRINI, V., MARTIN, J. C., MITREVA, M., MUZNY, D. M., SODERGREN, E. J., VERSALOVIC, J., WOLLAM, A. M., WORLEY, K. C., WORTMAN, J. R., YOUNG, S. K., ZENG, Q., AAGAARD, K. M., ABOLUDE, O. O., ALLEN-VERCOE, E., ALM, E. J., ALVARADO, L., ANDERSEN, G. L., ANDERSON, S., APPELBAUM, E., ARACHCHI, H. M., ARMITAGE, G., ARZE, C. A., AYVAZ, T., BAKER, C. C., BEGG, L., BELACHEW, T., BHONAGIRI, V., BIHAN, M., BLASER, M. J., BLOOM, T., BONAZZI, V., PAUL BROOKS, J., BUCK, G. A., BUHAY, C. J., BUSAM, D. A., CAMPBELL, J. L., CANON, S. R., CANTAREL, B. L., CHAIN, P. S. G., CHEN, I. M. A., CHEN, L., CHHIBBA, S., CHU, K., CIULLA, D. M., CLEMENTE, J. C., CLIFTON, S. W., CONLAN, S., CRABTREE, J., CUTTING, M. A., DAVIDOVICS, N. J., DAVIS, C. C., DESANTIS, T. Z., DEAL, C., DELEHAUNTY, K. D., DEWHIRST, F. E., DEYCH, E., DING, Y., DOOLING, D. J., DUGAN, S. P., MICHAEL DUNNE, W., SCOTT DURKIN, A., EDGAR, R. C., ERLICH, R. L., FARMER, C. N., FARRELL, R. M., FAUST, K., FELDGARDEN, M., FELIX, V. M., FISHER, S., FODOR, A. A., FORNEY, L. J., FOSTER, L., DI FRANCESCO, V., FRIEDMAN, J., FRIEDRICH, D. C., FRONICK, C. C., FULTON, L. L., GAO, H., GARCIA, N., GIANNOUKOS, G., GIBLIN, C., GIOVANNI, M. Y., GOLDBERG, J. M., GOLL, J., GONZALEZ, A., GRIGGS, A., et al. 2012. Structure, function and diversity of the healthy human microbiome. *Nature,* 486**,** 207-214.

KE, S., WEISS, S. T. & LIU, Y.-Y. 2022. Dissecting the role of the human microbiome in COVID-19 via metagenome-assembled genomes. *Nature Communications,* 13.

KRISTENSEN, J. M., NIERYCHLO, M., ALBERTSEN, M. & NIELSEN, P. H. 2020. Bacteria from the Genus Arcobacter Are Abundant in Effluent from Wastewater Treatment Plants. *Appl Environ Microbiol,* 86.

LI, P., CHANG, X., CHEN, X., WANG, C., SHANG, Y., ZHENG, D. & QI, K. 2022. Early-life antibiotic exposure increases the risk of childhood overweight and obesity in relation to dysbiosis of gut microbiota: a birth cohort study. *Annals of Clinical Microbiology and Antimicrobials,* 21.

LU, Y., REDLINGER, T. E., AVITIA, R., GALINDO, A. & GOODMAN, K. 2002. Isolation and genotyping of Helicobacter pylori from untreated municipal wastewater. *Appl Environ Microbiol,* 68**,** 1436-9.

MCILROY, S. J., ALBERTSEN, M., ANDRESEN, E. K., SAUNDERS, A. M., KRISTIANSEN, R., STOKHOLM-BJERREGAARD, M., NIELSEN, K. L. & NIELSEN, P. H. 2014. 'Candidatus Competibacter'-lineage genomes retrieved from metagenomes reveal functional metabolic diversity. *The ISME Journal,* 8**,** 613-624.

MCLELLAN, S. L., HUSE, S. M., MUELLER-SPITZ, S. R., ANDREISHCHEVA, E. N. & SOGIN, M. L. 2010. Diversity and population structure of sewage-derived microorganisms in wastewater treatment plant influent. *Environmental Microbiology,* 12**,** 378-392.

NAUD, S., BELLALI, S., ANANI, H., LO, C. I., YACOUBA, A., TIDJANI ALOU, M., ARMSTRONG, N., BONVALET, M., ZITVOGEL, L., RAOULT, D. & LAGIER, J. C. 2021. Luxibacter massiliensis gen. nov., sp. nov., a new bacterium isolated from the human gut microbiota. *New Microbes New Infect,* 40**,** 100850.

NIERYCHLO, M., ANDERSEN, K. S., XU, Y., GREEN, N., JIANG, C., ALBERTSEN, M., DUEHOLM, M. S. & NIELSEN, P. H. 2020. MiDAS 3: An ecosystem-specific reference database, taxonomy and knowledge platform for activated sludge and anaerobic digesters reveals species-level microbiome composition of activated sludge. *Water Res,* 182**,** 115955.

PETZOLDT, D., BREVES, G., RAUTENSCHLEIN, S. & TARAS, D. 2016. Harryflintia acetispora gen. nov., sp. nov., isolated from chicken caecum. *Int J Syst Evol Microbiol,* 66**,** 4099-4104.

PFEIFFER, N., DESMARCHELIER, C., BLAUT, M., DANIEL, H., HALLER, D. & CLAVEL, T. 2012. Acetatifactor muris gen. nov., sp. nov., a novel bacterium isolated from the intestine of an obese mouse. *Archives of Microbiology,* 194**,** 901-907.

QIN, J., LI, R., RAES, J., ARUMUGAM, M., BURGDORF, K. S., MANICHANH, C., NIELSEN, T., PONS, N., LEVENEZ, F., YAMADA, T., MENDE, D. R., LI, J., XU, J., LI, S., LI, D., CAO, J., WANG, B., LIANG, H., ZHENG, H., XIE, Y., TAP, J., LEPAGE, P., BERTALAN, M., BATTO, J.-M., HANSEN, T., LE PASLIER, D., LINNEBERG, A., NIELSEN, H. B., PELLETIER, E., RENAULT, P., SICHERITZ-PONTEN, T., TURNER, K., ZHU, H., YU, C., LI, S., JIAN, M., ZHOU, Y., LI, Y., ZHANG, X., LI, S., QIN, N., YANG, H., WANG, J., BRUNAK, S., DORÉ, J., GUARNER, F., KRISTIANSEN, K., PEDERSEN, O., PARKHILL, J., WEISSENBACH, J., BORK, P., EHRLICH, S. D. & WANG, J. 2010. A human gut microbial gene catalogue established by metagenomic sequencing. *Nature,* 464**,** 59-65.

QIU, X., MACCHIETTO, M. G., LIU, X., LU, Y., MA, Y., GUO, H., SAQUI-SALCES, M., BERNLOHR, D. A., CHEN, C., SHEN, S. & CHEN, X. 2020. Identification of gut microbiota and microbial metabolites regulated by an antimicrobial peptide lipocalin 2 in high fat diet-induced obesity. *International Journal of Obesity,* 45, 143-154.

SCHWIERTZ, A., HOLD, G. L., DUNCAN, S. H., GRUHL, B., COLLINS, M. D., LAWSON, P. A., FLINT, H. J. & BLAUT, M. 2002. Anaerostipes caccae gen. nov., sp. nov., a New Saccharolytic, Acetate-utilising, Butyrate-producing Bacterium from Human Faeces. *Systematic and Applied Microbiology,* 25, 46-51.

SHETTY, S. A., ZUFFA, S., BUI, T. P. N., AALVINK, S., SMIDT, H. & DE VOS, W. M. 2018. Reclassification of Eubacterium hallii as Anaerobutyricum hallii gen. nov., comb. nov., and description of Anaerobutyricum soehngenii sp. nov., a butyrate and propionate-producing bacterium from infant faeces. *International Journal of Systematic and Evolutionary Microbiology,* 68, 3741-3746.

SHKOPOROV, A. N., CHAPLIN, A. V., SHCHERBAKOVA, V. A., SUZINA, N. E., KAFARSKAIA, L. I., BOZHENKO, V. K. & EFIMOV, B. A. 2016. Ruthenibacterium lactatiformans gen. nov., sp. nov., an anaerobic, lactate-producing member of the family Ruminococcaceae isolated from human faeces. *Int J Syst Evol Microbiol,* 66, 3041-3049.

SOH, M., MIYAKE, S., LIM, A., DING, Y. & SEEDORF, H. 2019. Schaedlerella arabinosiphila gen. nov., sp. nov., a D-arabinose-utilizing bacterium isolated from faeces of C57BL/6J mice that is a close relative of Clostridium species ASF 502. *Int J Syst Evol Microbiol,* 69, 3616-3622.

TAKADA, T., KURAKAWA, T., TSUJI, H. & NOMOTO, K. 2013. Fusicatenibacter saccharivorans gen. nov., sp. nov., isolated from human faeces. *Int J Syst Evol Microbiol,* 63, 3691-3696.

WU, L., NING, D., ZHANG, B., LI, Y., ZHANG, P., SHAN, X., ZHANG, Q., BROWN, M. R., LI, Z., VAN NOSTRAND, J. D., LING, F., XIAO, N., ZHANG, Y., VIERHEILIG, J., WELLS, G. F., YANG, Y., DENG, Y., TU, Q., WANG, A., ACEVEDO, D., AGULLO-BARCELO, M., ALVAREZ, P. J. J., ALVAREZ-COHEN, L., ANDERSEN, G. L., DE ARAUJO, J. C., BOEHNKE, K. F., BOND, P., BOTT, C. B., BOVIO, P., BREWSTER, R. K., BUX, F., CABEZAS, A., CABROL, L., CHEN, S., CRIDDLE, C. S., DENG, Y., ETCHEBEHERE, C., FORD, A., FRIGON, D., SANABRIA, J., GRIFFIN, J. S., GU, A. Z., HABAGIL, M., HALE, L., HARDEMAN, S. D., HARMON, M., HORN, H., HU, Z., JAUFFUR, S., JOHNSON, D. R., KELLER, J., KEUCKEN, A., KUMARI, S., LEAL, C. D., LEBRUN, L. A., LEE, J., LEE, M., LEE, Z. M. P., LI, Y., LI, Z., LI, M., LI, X., LING, F., LIU, Y., LUTHY, R. G., MENDONÇA-HAGLER, L. C., DE MENEZES, F. G. R., MEYERS, A. J., MOHEBBI, A., NIELSEN, P. H., NING, D., OEHMEN, A., PALMER, A., PARAMESWARAN, P., PARK, J., PATSCH, D., REGINATTO, V., DE LOS REYES, F. L., RITTMANN, B. E., NOYOLA, A., ROSSETTI, S., SHAN, X., SIDHU, J., SLOAN, W. T., SMITH, K., DE SOUSA, O. V., STAHL, D. A., STEPHENS, K., TIAN, R., TIEDJE, J. M., TOOKER, N. B., TU, Q., VAN NOSTRAND, J. D., DE LOS COBOS VASCONCELOS, D., VIERHEILIG, J., WAGNER, M., WAKELIN, S., WANG, A., WANG, B., WEAVER, J. E., et al. 2019. Global diversity

and biogeography of bacterial communities in wastewater treatment plants. *Nature Microbiology,* 4**,** 1183-1195.

YATSUNENKO, T., REY, F. E., MANARY, M. J., TREHAN, I., DOMINGUEZ-BELLO, M. G., CONTRERAS, M., MAGRIS, M., HIDALGO, G., BALDASSANO, R. N., ANOKHIN, A. P., HEATH, A. C., WARNER, B., REEDER, J., KUCZYNSKI, J., CAPORASO, J. G., LOZUPONE, C. A., LAUBER, C., CLEMENTE, J. C., KNIGHTS, D., KNIGHT, R. & GORDON, J. I. 2012. Human gut microbiome viewed across age and geography. *Nature,* 486**,** 222-227.

ZOU, Y., XUE, W., LIN, X., LV, M., LUO, G., DAI, Y., SUN, H., LIU, S.-W., SUN, C.-H., HU, T. & XIAO, L. 2021. Butyribacter intestini gen. nov., sp. nov., a butyric acid-producing bacterium of the family Lachnospiraceae isolated from human faeces, and reclassification of Acetivibrio ethanolgignens as Acetanaerobacter ethanolgignens gen. nov., comb. nov. *Systematic and Applied Microbiology,* 44.
